# Multiple Longitudinal Tracts in the Cephalopod Arm Sensorimotor System

**DOI:** 10.1002/cne.70165

**Published:** 2026-05-05

**Authors:** Cassady S. Olson, Clifton W. Ragsdale

**Affiliations:** ^1^ Committee on Computational Neuroscience University of Chicago Chicago Illinois USA; ^2^ Department of Neurobiology University of Chicago Chicago Illinois USA

**Keywords:** *Doryteuthis pealeii*, *Euprymna berryi*, muscular hydrostat, neural circuitry, neuroethology, *Octopus bimaculoides*, sensorimotor control

## Abstract

Octopuses have an incredibly rich behavioral repertoire, exhibiting complex motor acts that require the coordination of eight highly flexible arms, each with hundreds of suckers. These movements are controlled by an axial nerve cord (ANC), analogous to the spinal cord, situated in the center of the arm musculature. The ANC has a cell body layer which forms a U‐shape around its neuropil and is capped aborally, or opposite the sucker, by the cerebrobrachial tract (CBT), a massive fiber bundle known to interconnect the arms and the brain. In vertebrate spinal cords, in addition to the major fiber tracts that interconnect the brain and spinal cord, there are spinospinal connectives that coordinate complex motor behaviors across the appendages. Here, we asked with tract‐tracing and immunohistochemistry, whether an octopus arm's ANC might also have intrinsic longitudinal connections for coordinated arm and sucker movements. We found that the ANC neuropil is enriched in longitudinal fibers. These fibers form distinct tracts, two within the oral (sucker‐side) neuropil and two in the aboral (brachial‐side) neuropil. In addition, the CBT itself demonstrates four major subtracts, and DiI labeling and dextran tracing suggest that (1) the CBT also carries arm‐intrinsic longitudinal connections and (2) the CBT and the neuropil tracts can be subcategorized into those that primarily connect with the sucker and those that serve the arm musculature. We also examined the organization of fiber tracts in the ANC of the arms and tentacles of two species of squid, establishing that an aboral, extra‐neuropil tract is a shared feature across all cephalopod species studied. In addition, the squids have an oral longitudinal tract, though its positioning and size varied with species and appendage. In sum, these findings describe the neural substrate for coordinating motor behaviors along the length of a cephalopod appendage.

## Graphical abstract

Octopuses have a rich behavioral repertoire, coordinating complex movements along the length of an arm. The neural circuits controlling these behaviors are poorly understood. We employ tract‐tracing to investigate longitudinal tracts in the octopus arm. We describe multiple tracts within the axial nerve cord, which mediate longer range arm movements.

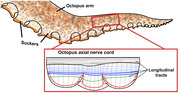

## 1 Introduction

Octopuses possess a motor control challenge of enormous complexity (Hochner et al. 2023; Olson and Ragsdale 2023). Each of the hydrostatic arms moves in near infinite degrees of freedom, and each arm is lined with hundreds of suckers, which also move independently (Kier and Stella 2007; Kennedy et al. 2020; Röckner et al. 2023). Critical behaviors from locomotion to feeding require the octopus to coordinate motor activity along the length of an arm and between arms (Gutfreund et al. 1998; Sumbre et al. 2006; Kennedy et al. 2020; Bidel et al. 2022; Bennice et al. 2025). Furthermore, many of these behaviors require the coordination of sucker movements and the collaboration of motor activity between the suckers and the arm (Grasso 2008; Kennedy et al. 2020; Buresch et al. 2022, 2024; Bennice et al. 2025).

**FIGURE 1:**
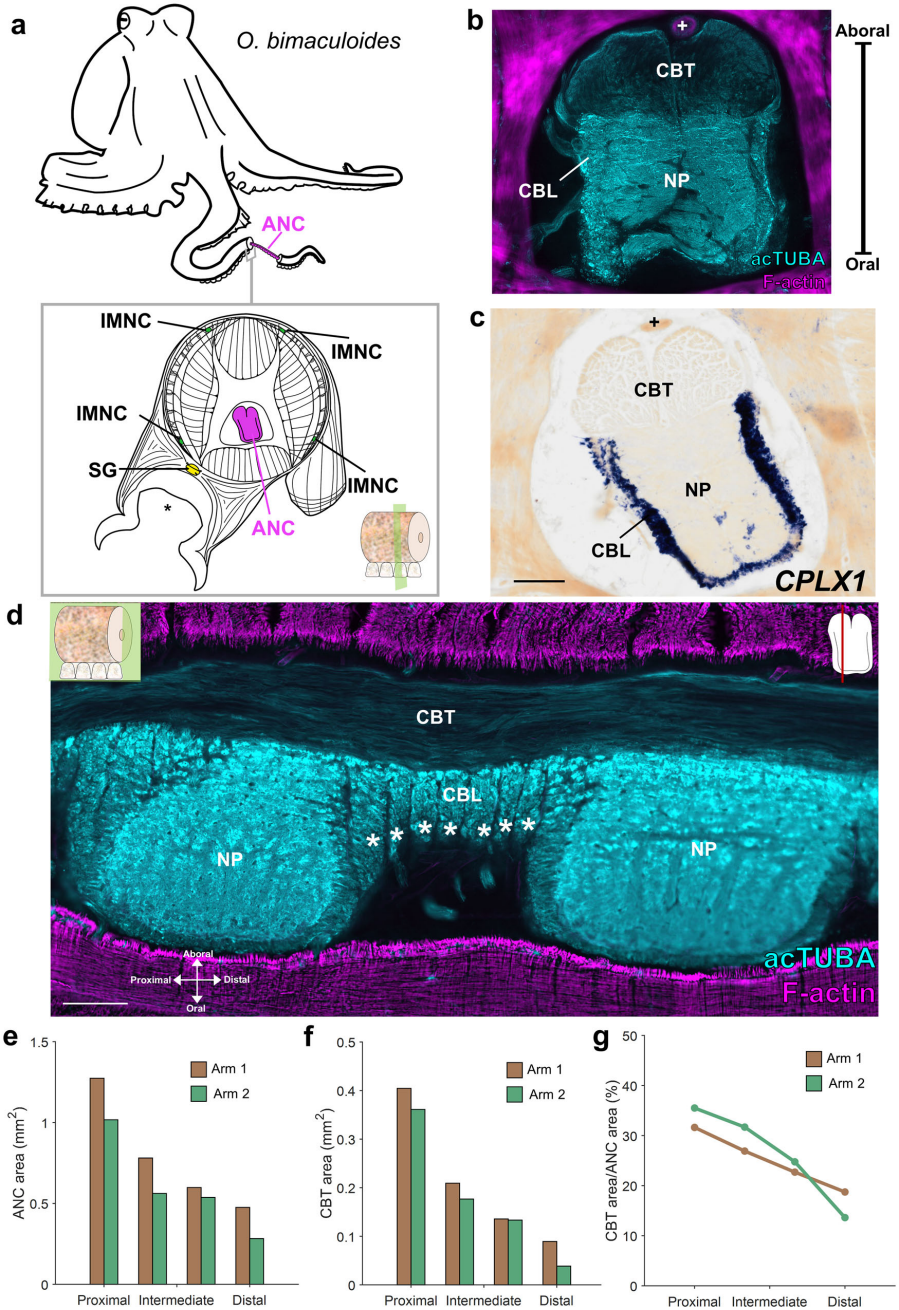
Cerebrobrachial tract composition and proximal‐distal variation. (a) Cartoon of *Octopus bimaculoides* and diagram of a transverse cross section of octopus arm with the major components of the arm nervous system highlighted: magenta, axial nerve cord (ANC); green, intramuscular nerve cord (IMNC); yellow, sucker ganglion (SG). Key indicates transverse plane of section. * denotes acetabulum of the sucker. (b) Transverse section of the ANC stained for acetylated α‐tubulin (acTUBA, cyan) and F‐actin (magenta). The cerebrobrachial tract (CBT) is aboral (away from the sucker) to the cell body layer (CBL) and neuropil (NP) and is composed of acTUBA‐positive fibers. The brachial artery, denoted by +, is situated in between the two CBT trunks. (c) In situ hybridization (ISH) for complexin 1 (*CPLX1)* gene expression in a transverse section of the ANC labels neuronal cell bodies. Notably, the CBT is devoid of labeling. + denotes brachial artery. Scale bar: 250 µm. (d) Longitudinal section of the ANC stained for acTUBA (cyan) and F‐actin (magenta). CBT fibers are oriented longitudinally. CBL segments, denoted by white asterisks, are readily apparent in this preparation. Keys indicate plane of section. Scale bar: 250 µm. (e, f) Cross sectional area of the ANC (e) and (f) the CBT decreases from proximal to distal. The total cross‐sectional area of the ANC = CBL area + NP area + CBT area. (g) The proportion of total ANC area occupied by the CBT decreases from proximal to distal. acTUBA, acetylated α‐tubulin; ANC, axial nerve cord; CBL, cell body layer; CBT, cerebrobrachial tract; IMNC, intramuscular nerve cord; NP, neuropil; SG, sucker ganglion.

Neural control for arm and sucker movements arises from the extensive nervous system embedded in the arm itself and from the intermediate motor centers in the octopus brain (Figure 1a; Graziadei 1971; Young 1971; Hochner et al. 2023; Olson and Ragsdale 2023). Each arm houses an axial nerve cord (ANC), analogous to the vertebrate spinal cord, situated in the center of the brachial musculature (Graziadei 1971; Olson and Ragsdale 2023; Neacsu and Crook 2024; Olson, Schulz, et al. 2025). The ANC contains a cell body layer (CBL) that wraps around its neuropil (NP) (Figure 1b,c). The CBL comprises repeated segments down the long axis of the arm (Figure 1d), with the cell bodies issuing their processes directly into the NP adjacent to each segment (Olson, Schulz, et al. 2025). Orthogonal to this architecture, the CBL and NP can be split into an oral (towards to the sucker) sucker territory and an aboral (away from the sucker) brachial territory (Rowell 1963; Olson, Schulz, et al. 2025). The oral sucker territory of the ANC forms local enlargements for each sucker (Rossi and Graziadei 1954; Olson, Schulz, et al. 2025), and stimulation of this territory elicits sucker movements (Rowell 1963). Within these enlargements, nerves connecting with the sucker epithelium maintain their spatial positioning, thereby forming an internal suckerotopy (Olson, Schulz, et al. 2025). Within the aboral NP, nerves corresponding to the brachial musculature enter in the septa between segments and bifurcate to branch over adjoining segments (Olson, Schulz, et al. 2025). Stimulation of this aboral, brachial territory causes movements of the arm as a whole (Rowell 1963). While ANC segments in the CBL and the division for brachial and sucker territories provide the structure for parsing the arm into smaller units for local control, they do not account for how movements of the suckers and the arm itself are coordinated over a distance.

In vertebrate nervous systems, major fiber tracts serve to interconnect the brain and spinal cord, allowing for the control and coordination of complex motor acts. These tracts can be divided into ascending sensory tracts, transmitting sensory input from the periphery, and descending motor tracts, relaying motor commands to spinal cord motor neurons. Analogously, the octopus ANC features a cerebrobrachial tract (CBT) situated aboral to the CBL and NP (Graziadei 1971; Imperadore et al. 2019; Zullo et al. 2019; Neacsu and Crook 2024). The CBT comprises two massive trunks of longitudinally running fibers that interconnect the arms and the brain (Graziadei 1971; Imperadore et al. 2019; Zullo et al. 2019). As the ANC ascends towards the brain, the CBT coalesces into a single trunk (Graziadei 1971). Classic studies have noted a parcellation of CBT fibers based on size, though their relative functions were unknown (Graziadei 1971). Recent electrophysiological experiments posit that the CBT transmits motor signals *en passant*, allowing for an interplay between signals from the brain and ANC circuitry (Zullo et al. 2019), and recent tracing studies showcase fibers deflecting from the CBT toward the NP (Imperadore et al. 2019). These studies are, however, few in number, and there is little experimental evidence for how longitudinally running tracts in the CBT are organized.

The vertebrate spinal cord also contains spinospinal connections, which are particularly prominent, and in fact quite extensive, in four‐legged carnivores (Sherrington and Laslett 1903). These spinospinal fibers travel in spinal cord white matter, thereby invading the major ascending and descending fascicles. In the octopus system, classic studies suggest shorter interconnecting systems within the NP of the ANC, which most certainly assist in behaviors coordinating suckers and arm movements along a single arm (Graziadei 1971). The relative positioning and trajectories of these inter‐NP tracts are not known.

Here, we employed tract‐tracing methods and immunohistochemistry to investigate the organization of CBT in *Octopus bimaculoides*. We also interrogated the ANC NP for additional longitudinal fiber tracts. Finally, we examined the organization of fiber tracts in the ANC of the arms and tentacles of two species of squid to ask what the shared features of coleoid appendage fiber tracts are. Our findings establish the presence of multiple longitudinal fiber tracts, in the CBT and both the oral and aboral NPs, that are strong candidates to mediate longer range movement coordination.

## 2 Materials and Methods

### 2.1 Animals

Aquatic Research Consultants, a field‐collection venture operated by Dr. Chuck Winkler, San Pedro, CA, supplied wild‐caught *O. bimaculoides* adults (*Obi, n* = 17) of both sexes and at least six months of age. Octopuses were individually housed in 20‐gallon saltwater tanks equipped with an UV light, carbon particle filter, and an aquarium bubbler. Fiddler crabs, bivalves or frozen shrimp were fed to the octopuses daily. The artificial seawater (ASW) was prepared by adding pharmaceutically pure sea salt (33 g/L; Tropic Marin “classic”, Wartenberg, Germany) to deionized water. Wild‐caught adult *Doryteuthis pealeii* (*Dpe, n* = 3) and lab‐cultured adult *Euprymna berryi* (*Ebe, n* = 3) of both sexes were obtained at the Marine Biological Laboratory, Woods Hole, MA.

These cephalopod experiments were performed in compliance with the EU Directive 2010/63/EU guidelines on cephalopod use, University of Chicago Animal Resources Center oversight, and the University of Chicago Institutional Animal Care and Use Committee (Fiorito et al. 2015; Lopes et al. 2017; Copio et al. 2026).

### 2.2 Tissue Collection

Octopuses were deeply anesthetized in 4% ethanol/ASW (EtOH/ASW, *n* = 9) and trans‐orbitally perfused with 4% paraformaldehyde/phosphate‐buffered saline solution (PFA/PBS, pH 7.4) delivered via a peristaltic pump (Cole Parmer Masterflex) through a 21½ gauge needle (Becton Dickinson). The left and right white bodies, a hematopoietic tissue, were each targeted multiple times throughout the procedure (Wang and Ragsdale 2024; Olson, Moorjani, et al. 2025). Arms were dissected and stored in 4% PFA/PBS overnight at 4°C. Specimens were then either cut into 2–4 cm pieces for immediate processing or stored in diethyl pyrocarbonate (DEPC)‐treated PBS at 4°C.

Squid were kept in circulating, filtered containment tanks for several days before being deeply anesthetized in 7.5% MgCl_2_/ASW and dissected (*Dpe*, *n* = 3; *Ebe*, *n* = 3). Arm crowns were immersion‐fixed in 4% PFA/PBS. Arms and tentacles were dissected and cut into 2–4 cm pieces for further processing.

### 2.3 Slide Preparation

Octopus and squid tissue blocks were placed in 30% sucrose/4%PFA/PBS at 4°C until saturated (3–5 days), rinsed with 30% sucrose/PBS, and infiltrated with 10% gelatin/30% sucrose/PBS for 1 h at 50°C. The tissue now embedded in 10% gelatin/30% sucrose/PBS was post‐fixed in 30% sucrose/4%PFA/PBS before storage at −80°C. Serial arm sections were cut at 28–50‐µm thickness on a freezing microtome (Leica SM2000R) and collected in DEPC‐PBS. Sections were mounted and dried on charged, hydrophilic glass slides (TruBond380, Newcomer Supply, Middleton, WI), then stored at −80°C until further processing.

### 2.4 In Situ Hybridization

In situ hybridization with complexin 1 (*CPLX1)*, a pan‐neuronal marker for the presynaptic apparatus, was utilized to demonstrate the distribution of neuronal cell bodies in the ANC. To generate antisense riboprobes for *CPLX1*, plasmids were linearized through SpeI (New England Biolabs, Cat#: 50811989) restriction enzyme digestion to make template. Following phenol‐chloroform extraction of the template, antisense digoxigenin (DIG)‐labeled riboprobes (Sigma‐Aldrich, Cat#: 11277073910) were transcribed with T7 RNA polymerase (New England Biolabs). After transcription, residual template was digested with RNase‐free DNase I (Sigma‐Aldrich) at 37°C for 15 min. Riboprobes were ethanol‐precipitated and stored in 100 µL of DEPC‐H2O at −20°C until use. The cloning for *CPLX1* was previously reported (Olson, Schulz, et al. 2025)

Slides of sectioned octopus tissue were equilibrated to room temperature, then post‐fixed in mailers for 15 min in 4% PFA/PBS, washed 3 × 15 min (3 times for 15 min each) in DEPC‐PBS and incubated at 37°C for 15 min in proteinase K solution (Sigma‐Aldrich; 1.45 µg proteinase K per milliliter of 100 mM Tris‐HCl [pH 8.0], 50 mM EDTA [pH 8.0]). Following a 15‐min post‐fix in 4% PFA/PBS, slides were washed 3 × 15 min in DEPC‐PBS and acclimated to 72°C for 1 h in hybridization solution (50% formamide, 5x SSC, 1% SDS, 200 µg/mL heparin, 500 µg /mL yeast RNA). Slides were transferred to mailers with 1–2 µg antisense riboprobe in 15 mL hybridization solution and incubated overnight at 72°C. The next day, slides were treated 3 × 45 min in preheated Solution X (50% formamide, 5x SSC, 1% SDS) at 72°C. Slides were washed 3 × 15 min in room temperature TBST (Tris‐buffered saline with 1% Tween 20) and blocked at room temperature for 1 h in 10% DIG buffer (Roche) in TBST.

Anti‐DIG Fab fragments conjugated to alkaline phosphatase (Sigma‐Aldrich, Cat#: 11093274910; RRID: AB_514497) were pre‐absorbed with octopus embryo powder in 1% DIG buffer in TBST for at least one hour. Slides were then incubated on a rocker overnight at 4°C in preabsorbed antibody diluted to a final concentration of 1:5000 in 10% DIG buffer in TBST.

The next day, slides were quickly rinsed once, then for 3 × 15 min, then for 2 × 1 h in TBST. Slides were placed in freshly prepared NTMT (100 mM Tris‐HCl [pH 9.5], 100 mM NaCl, 50 mM MgCl2, 1% Tween 20) for 10 min. For the color reaction, slides were incubated in 0.35 mg/mL nitro blue tetrazolium (NBT, stock: 100 mg/mL in 70% dimethyl formamide/30% DEPC‐H20, Gold Biotechnology, St. Louis, MO) and 0.175 mg/mL 5‐bromo‐4‐chloro‐3‐indolyl phosphate (BCIP, stock: 50 mg/mL in 100% dimethyl formamide, Gold Biotechnology, St. Louis, MO) in NTMT. Color development proceeded at room temperature and was monitored for a maximum of 5 days. When the reaction appeared complete, slides were washed in TBST overnight and dehydrated through a series of ethanol washes (2 × 70%, 2 × 95 %, each for 30 s; then 2 × 100% for 1 min each) into Histoclear (3 × 1 min), and coverslipped with Eukitt (Sigma‐Aldrich).

### 2.5 Section and Whole Mount Immunohistochemistry

Neuronal processes in the octopus arm and the squid arm and tentacle were labeled with mouse monoclonal antibody 6–11B‐1 (1:500 dilution of ascites fluid; Sigma‐Aldrich, Cat#: T6793; RRID: AB_477585) and mouse monoclonal antibody SMI‐31 (1:500 dilution; BioLegend, Cat# 801601; RRID: AB_2564641). Clone 6–11B‐1 was isolated following immunization with sea urchin sperm flagella protein preparations (Piperno and Fuller 1985). It recognizes an acetylated α‐tubulin (acTUBA) epitope found broadly but not universally across microtubules and has been extensively employed to identify axon tracts in vertebrate and invertebrate nervous systems (Chitnis and Kuwada 1990; LeDizet and Piperno 1991; Shigeno and Yamamoto 2002; Baratte and Bonnaud 2009; Olson, Schulz, et al. 2025; Olson, Moorjani, et al. 2025). Clone SMI‐31 reacts with a phosphorylated epitope of neurofilament in mammals and neurofilament 220 in squid (Sternberger and Sternberger 1983; Grant et al. 1995; Grant and Pant 2016), and it has been employed successfully in octopus arm tissue (Olson, Schulz, et al. 2025). The secondary Alexa Fluor 488 AffiniPure Goat Anti‐Mouse IgG (Cat#: 115‐545‐003; RRID: AB_2338840) and Cy3 AffiniPure Donkey Anti‐Mouse IgG (Cat#: 715‐165‐151; RRID: AB_2315777) were purchased from Jackson ImmunoResearch (West Grove, PA) and utilized at 1:500 dilutions.

For section immunohistochemistry, slides were rinsed 3 × 10 min in DEPC‐PBS containing 1% Tween 20 (PBST) and incubated for 30 min in a proteinase K solution (Sigma‐Aldrich, Cat#: 03115828001; 19.4 µg proteinase K per milliliter of PBST). Slides were post‐fixed with 4% PFA/PBS for 15 min, washed 3 × 30 min in PBST, blocked in 10% goat serum/PBST (Fisher Scientific, Cat#: 16210072) for 1 h and incubated in primary antibody diluted in 1% goat serum/PBST for 4 days at 4°C. After 3 × 30 min PBST washes, sections were incubated for two days at 4°C with secondary antibody along with 0.01 mg/mL 4′‐6‐diamidino‐2‐phenylindole (DAPI; Sigma‐Aldrich, Cat#: D9542) to label cell nuclei fluorescently and either 0.2 µL/mL phalloidin‐iFluor 594 (Abcam, Cat#: ab176757) or phalloidin‐iFluor 488 (Abcam, Cat#: ab176753) to label filamentous actin (F‐actin). Slides were washed 3 × 5 min with PBST and coverslipped with Fluoromount G (Southern Biotech, Birmingham, AL).

For whole mount immunohistochemistry, the following octopus arm blocks were prepared: 0.5–1 cm transverse slices, or longitudinally cut slices, or horizontally cut slices. Slices were washed 3 × 15 min in PBST, dehydrated in a graded methanol series (25%, 50%, 75% in PBST, each for 10 min), rinsed twice in 100% methanol for 10 min, and stored overnight at −20°C. The next day, slices were rehydrated in a graded methanol series (75%, 50%, 25% in PBST, each for 5 min) and rinsed twice in PBST for 5 min. Then, the tissue was incubated for 60 min at 37°C in a proteinase K solution (19.4 µg proteinase K per milliliter of PBST), post‐fixed for 15 min in 4% PFA/PBS, washed 3 × 15 min and 1 × 1 h in PBST, and blocked for an hour in 10% goat serum/PBST. Slices were incubated in primary antibody for 7 days at 37°C. Following primary incubation, tissue was rinsed 1x, then 3 × 15 min, then 5 × 1 h, and then overnight in PBST. Tissue was transferred to secondary antibody for seven days at 4°C. The tissue was rinsed with PBST 1x quickly, 3 × 15 min, 2 × 1 h, washed with DEPC‐PBS 3 × 15 min, and post‐fixed for four days at 4°C. Slices were then rinsed 3 × 15 min in PBS and cleared in a modified version of FRUIT (35%, 40%, 60%, 80%, and 100% FRUIT, each for 24 h; Hou et al. 2015). Slices were stored in 100% FRUIT at 4°C until imaged.

### 2.6 DiI Labeling

DiI crystals (Biotium, Cat#: 60016) were placed at various targets in the 0.5–1 cm transversely, horizontally and longitudinally cut fixed arm blocks. Slices were incubated in 1% PFA/PBS in the dark. Since the most appropriate incubation time was unknown, slices were checked weekly; then stopped once the labelling of connections to their targets were complete. This was as short as 7 days for short tracing distances and up to 6 months for longer distances. As in other systems (Godement et al. 1987), DiI in octopus elicits retrograde and anterograde cell labelling. Following DEPC‐PBS washes for 3 × 15 min, explants were cleared in a modified version of FRUIT (Hou et al. 2015).

### 2.7 Tracing

Adult *O. bimaculoides* (*n* = 10) were anesthetized in 2% EtOH/ASW, and the distal portion of a single arm was amputated with a razor blade. Each octopus experienced a maximum of two amputations. After its first amputation, the octopus was placed in a bucket of ASW to recover before being returned to its home tank. The octopus was monitored for 24 h to ensure a return to regular feeding. After any second amputation, the anesthetic solution was increased to 4% EtOH/ASW before perfusion.

The amputated arm was cut with a razor blade into 0.5–1 cm transverse slices. Slices were placed into 221 media with NuSerum (36% Leibovitz L15 media, 36% filtered ASW, 18% deionized water, 1% pen‐strep, 10% NuSerum) prior to injection. Each slice (*n* = 103) was injected with CF 488A Dye Dextran (Biotium, Cat#: 80110; 2% solution, 10 kD) using a 25‐ or 32‐gauge Hamilton syringe (Hamilton, model#: 7000.5), rinsed in filtered ASW, and incubated at room temperature in 221 media with NuSerum. In octopus, as in vertebrates, dextrans serve as retrograde and anterograde tracers (Reiner and Honig 2006; Olson, Schulz, et al. 2025). After 3 h, the slices were rinsed with filtered ASW and fixed in 4% PFA/PBS. Slices were gelatin‐embedded as described above, sectioned at 50 µm, and counterstained with phalloidin‐iFluor 594 (0.2 µL/mL in 1%PBST) and DAPI (0.01 mg/ml in 1% PBST) before imaging.

### 2.8 Imaging

The immunolabeled and tracing tissue was studied with a Zeiss Axioskop 50 upright microscope and a Leica MZ FLIII stereomicroscope, both outfitted with the Zeiss AxioCam digital camera and AxioVision 4.5 software system. Selected sections were also studied on a Leica SP5 Tandem Scanner Spectral 2‐photon confocal microscope (Leica Microsystems, Inc., Buffalo Grove, IL), a 3i Marianas Super‐resolution through Optical Reassignment (SoRa) Yokogawa‐type spinning disk confocal microscope (Intelligent Imaging Innovations, Denver, CO) with dual Prime 95B Illuminated Scientific CMOS (Teledyne Photometrics Vision Solutions, Tucson, AZ) run by Slidebook software (Intelligent Imaging Innovations, Denver, CO), or scanned with an Olympus VS200 Research Slide Scanner (Olympus/Evident, Center Valley, PA) with a Hamamatsu ORca‐Fusion camera (Hamamatsu Photonics, Skokie, IL). Whole mounts were imaged on a LaVision BioTec UltraMicroscope II (Miltenyi Biotec, Bergish Gladbach, Germany) run by ImSpector Pro v. 7_124 software (LaVision BioTec, Bielefeld, Germany). Collected images were corrected for contrast and brightness and false‐colored in FIJI (version 2.1.0/1.53c; National Institutes of Health [NIH]). Diagrams were drawn using GIMP v. 3.0.41.0.

### 2.9 CBT Proximal‐Distal Analysis

To study proximal‐distal variation in the CBT, four whole mount slices spanning the proximal‐distal axis of one arm from two different animals (Arms 1L and 3R) were immunostained for acTUBA. Transverse confocal stacks were imaged at 10× to capture the whole slice and at 20× to capture the CBT. Maximum projections of the CBT were used to determine CBT subdivisions. Maximum projections of the whole slice were used to determine area.

#### 2.9.1 CBT Subdivisions

Across all slices, discrete bundles of fibers within the CBT were outlined. Bundles that were consistent along the proximal‐distal axis and between arms were demarcated as the main subdivisions.

#### 2.9.2 CBT Area

The areas of the CBT and the ANC were calculated using the polygon tool in FIJI. To ensure the measurement was accurate, adhering to the contours of the amorphous octopus anatomy, tracing dots were placed as close to each other as possible. The area for the CBT was then compared to the area of the ANC (CBT/ANC).

## 3 Results

### 3.1 Organization of the CBT

Within the ANC of *O. bimaculoides*, the CBT comprises two trunks of longitudinally arranged nerve fascicles situated at the most aboral portion of the ANC (Figure 1b,d). The brachial artery marks the midline between the two trunks (Figure 1b; Graziadei 1971; Olson, Schulz, et al. 2025). As demonstrated by in situ hybridization with the pan‐neuronal marker *CPLX1* and with immunostaining for acTUBA, the CBT is devoid of neuronal cell bodies (Figure 1b–d). The arm tapers down the long axis, with the suckers, brachial musculature, and ANC all decreasing in size (Figure 1e; Olson, Schulz, et al. 2025). The area of the CBT likewise decreases from proximal to distal (Figure 1f). Moreover, the proportion of the total ANC area occupied by the CBT decreases from proximal to distal (Figure 1g). This decrease in fiber tract size is reminiscent of vertebrate spinal cord tracts, which are largest closest to the brain (Parent and Carpenter 1996), and presumably reflects the decrease in the number of fibers projecting to and from the brain as one moves away from the brain.

**FIGURE 2:**
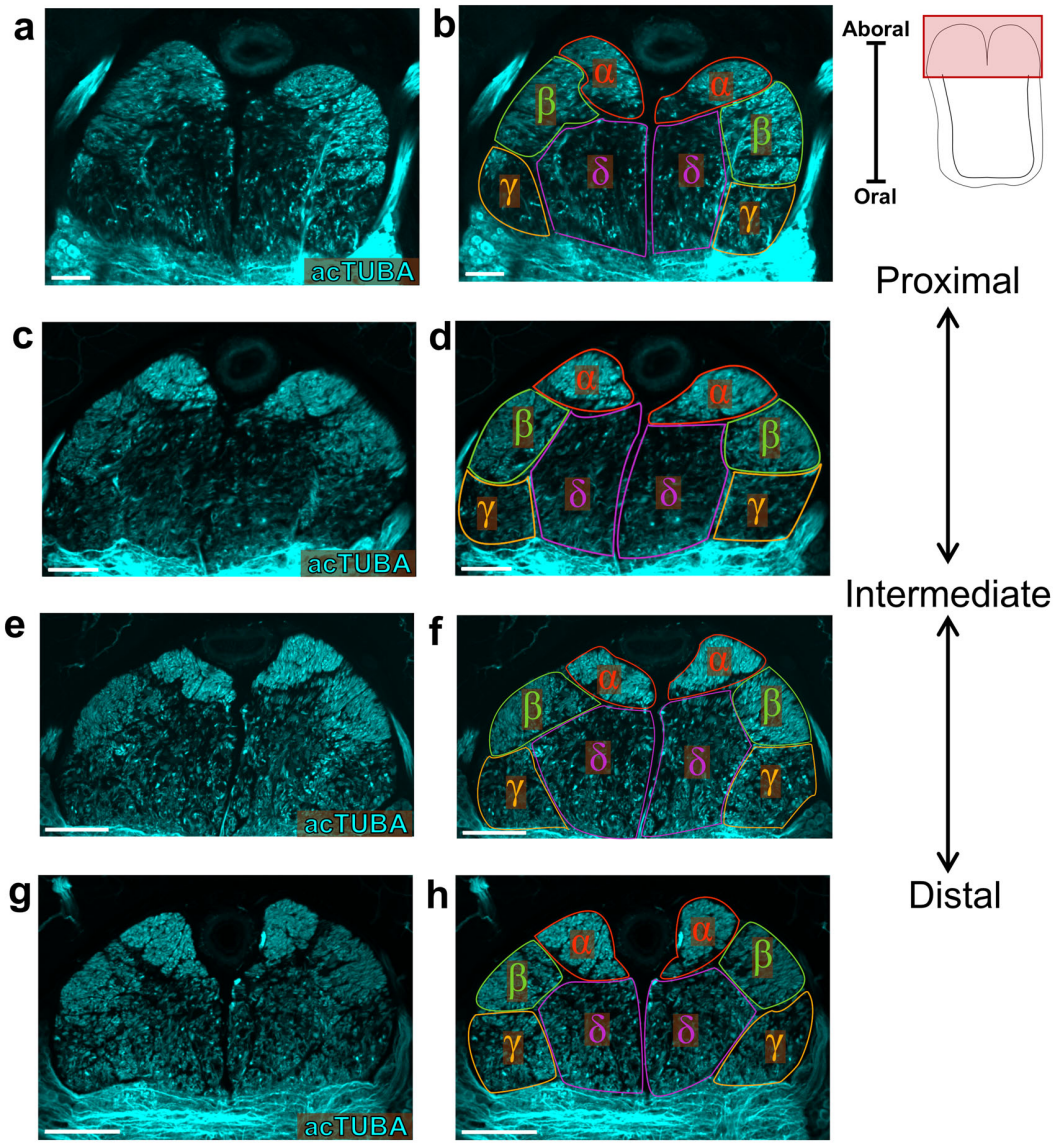
Subdivisions of the cerebrobrachial tract. (a–h) Maximum projections of the cerebrobrachial tract (CBT) in transverse wholemount slices from proximal (a, b) to distal (g, h) stained for acetylated α‐tubulin (acTUBA). The CBT can be subdivided into four major tracts which can be recognized at all proximal‐distal levels: α (red), β (green), γ (orange) and δ (magenta) tracts. Data used to define the tracts is presented in the left column (a, c, e, g). Tracts are outlined in the right column (b, d, f, h). Key on top right indicates field of view. Scale bars: 100 µm. acTUBA, acetylated α‐tubulin.

**FIGURE 3:**
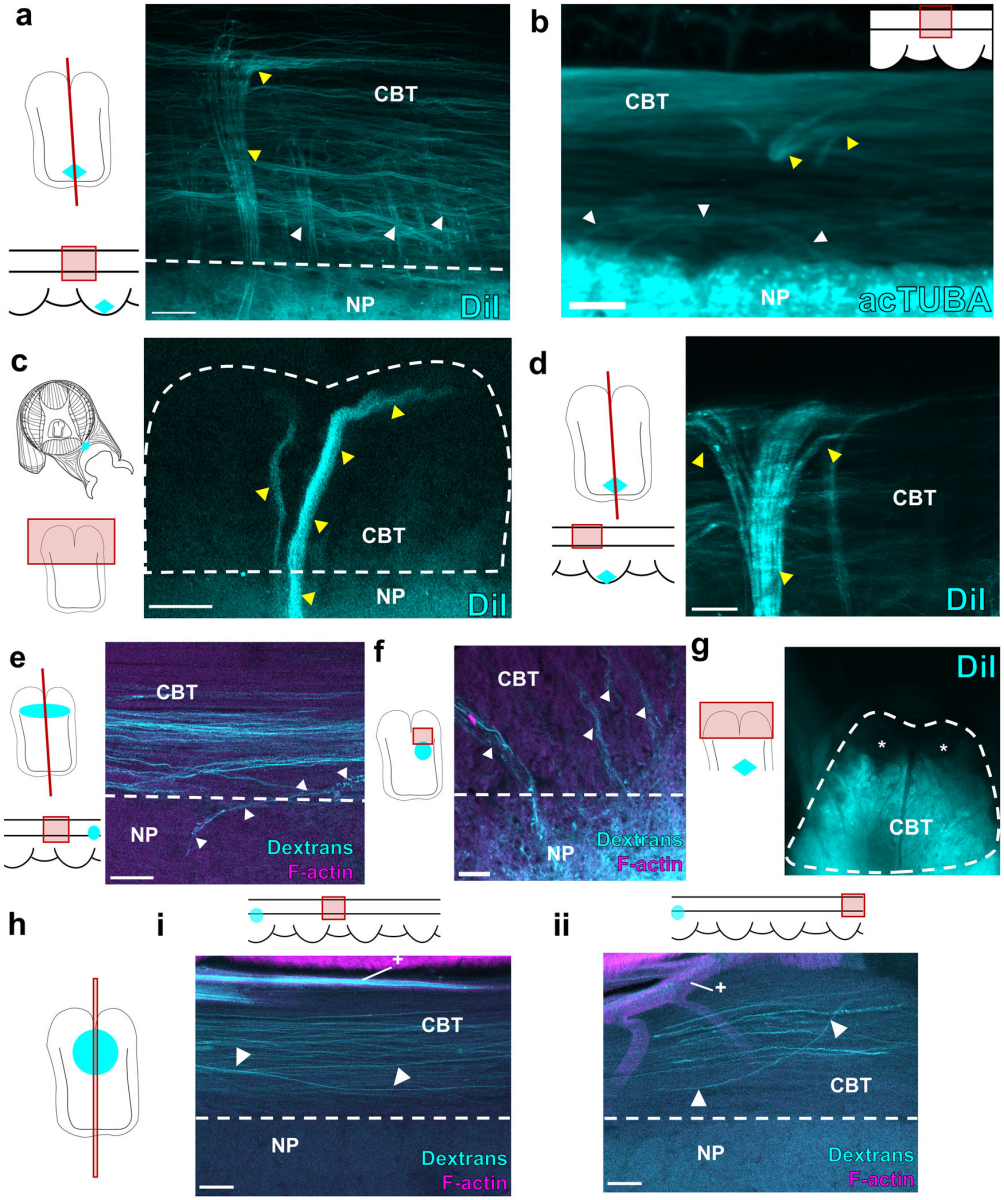
Cerebrobrachial tract and neuropil interactions. (a, b) The cerebrobrachial tract has many interactions with the NP. Yellow arrowheads point to large fascicles. White arrowheads mark small processes. (a) Maximum projection of the cerebrobrachial tract (CBT) in a longitudinal wholemount with DiI (cyan) crystal placed in oral neuropil (NP). Keys indicate planes of section and fields of view. Bold black lines in longitudinal key indicate the limits of the CBT. Diamonds in keys demonstrate location of DiI crystal. (b) Maximum projection of the CBT in a longitudinal wholemount immunostained for acetylated α‐tubulin (acTUBA). Inset demonstrates field of view. Scale bars: 100 µm. (c) CBT in a transverse wholemount with DiI (cyan) crystal placement in sucker musculature and including the sucker ganglion (diamond on key marks location of DiI crystal). Large processes that target the α tract are labeled (yellow arrowheads). Scale bar: 50 µm. (d) Maximum projection of the CBT in a longitudinal wholemount of ANC with DiI (cyan) crystal placed in oral neuropil (NP; diamonds on keys mark crystal placement). Connections to the α tract branch proximally and distally, indicated by yellow arrowheads. Scale bar: 100 µm. (e) Longitudinal section of an injection of dextran (cyan) into the CBT. White arrowheads point to projection from CBT that dips into the aboral NP. Keys indicate plane of section. Ovals on keys demonstrate the injection site. Scale bar: 100 µm. (f) Transverse section of an injection of dextran (cyan) into the aboral NP demonstrating connections to the CBT. Oval on key indicates injection site. Scale bar: 50 µm. (g) Transverse wholemount with DiI (cyan) crystal placement in the aboral neuropil. The β, γ, and δ tracts are labeled, but not the α tract, denoted with *. Diamond on key indicates location of crystal placement. (hi–ii) Longitudinal tracing following an injection of dextran into the CBT. Oval on key indicates location of injection site. Fascicles within the CBT travel a distance and rearrange. (i) Field of view four suckers away from the injection site. White arrowheads point to CBT fascicle that descends orally while remaining within the CBT. (ii) Field of view six suckers away from the injection site. White arrowheads point to CBT fascicle that ascends aborally. + denotes brachial artery. Scale bars: 100 µm. acTUBA, acetylated α‐tubulin; CBL, cell body layer; CBT cerebrobrachial tract; NP, neuropil.

Using transverse whole mounts immunostained for acTUBA, we identified subdivisions in the CBT trunks. Four distinct bundles could be recognized along the proximal‐distal axis in the two arms studied (α, β, γ, and δ tracts; Figure 2). These bundles are paired across the anterior‐posterior midline of the ANC. The α tracts are situated most aborally and are the most prominent in staining for acTUBA. The δ tracts are oral to the α tracts and hug the midline. The γ and β tracts are situated on the lateral sides of the CBT, with the γ tract positioned closest to the NP and the β tract located more aborally. These four main tracts demonstrate further sub‐compartments, such as those visible in the α tract on the distal slice (Figure 2g,h) or in the β tract on the proximal slice (Figure 2a,b). However, we could not reliably identify these additional subdivisions across all slices. This suggests either the fibers in the tracts reorganize along the proximal‐distal axis (see Figure 3h), or groups of fibers are specifically lost or gained along the length of the arm, or this staining is not a reliable method for identifying further subdivisions.

The CBT demonstrates many connections with the NP (Imperadore et al. 2019), with large processes targeting the aboral α tract and smaller processes targeting oral bundles (γ and δ tracts; Figure 3a,b). By placing DiI crystals in the sucker musculature (Figure 3c) or in the oral (sucker) territory of the ANC NP (Figure 3d), we elicited extensive labeling of fascicles targeting to the α tract. These connections primarily targeted the CBT trunk ipsilateral to the sucker, although fibers targeting the CBT trunk contralateral to the sucker were observed (Figure 3c). Once these processes reach the α tract, they split into proximal and distal branches, directed both towards and away from the brain (Figure 3d). In contrast, injections of dextran into the γ and δ tracts labelled projections dipping into the aboral, brachial NP (Figure 3e), and reciprocal injections into the aboral, brachial NP labelled projections targeting the γ and δ tracts (Figure 3f). Finally, DiI crystal placement into the aboral brachial NP demonstrates labeling in β, δ, and γ tracts, but not the α tract (Figure 3g). These results suggest that the β, δ, and γ tracts correspond to connections primarily related to the brachial musculature, though connections with the sucker could also be labeled as fibers of passage. Once within the CBT, some fibers maintain their position (Figure 3e,h). Others re‐sort, descending orally or ascending aborally at substantial distances from the injection site (Figure 3h). This kind of repositioning has been seen in vertebrate fiber tracts connecting the retina to the brain (Chan and Guillery 1994).

### 3.2 Longitudinal Tracts in *O. bimaculoides* NP

**FIGURE 4:**
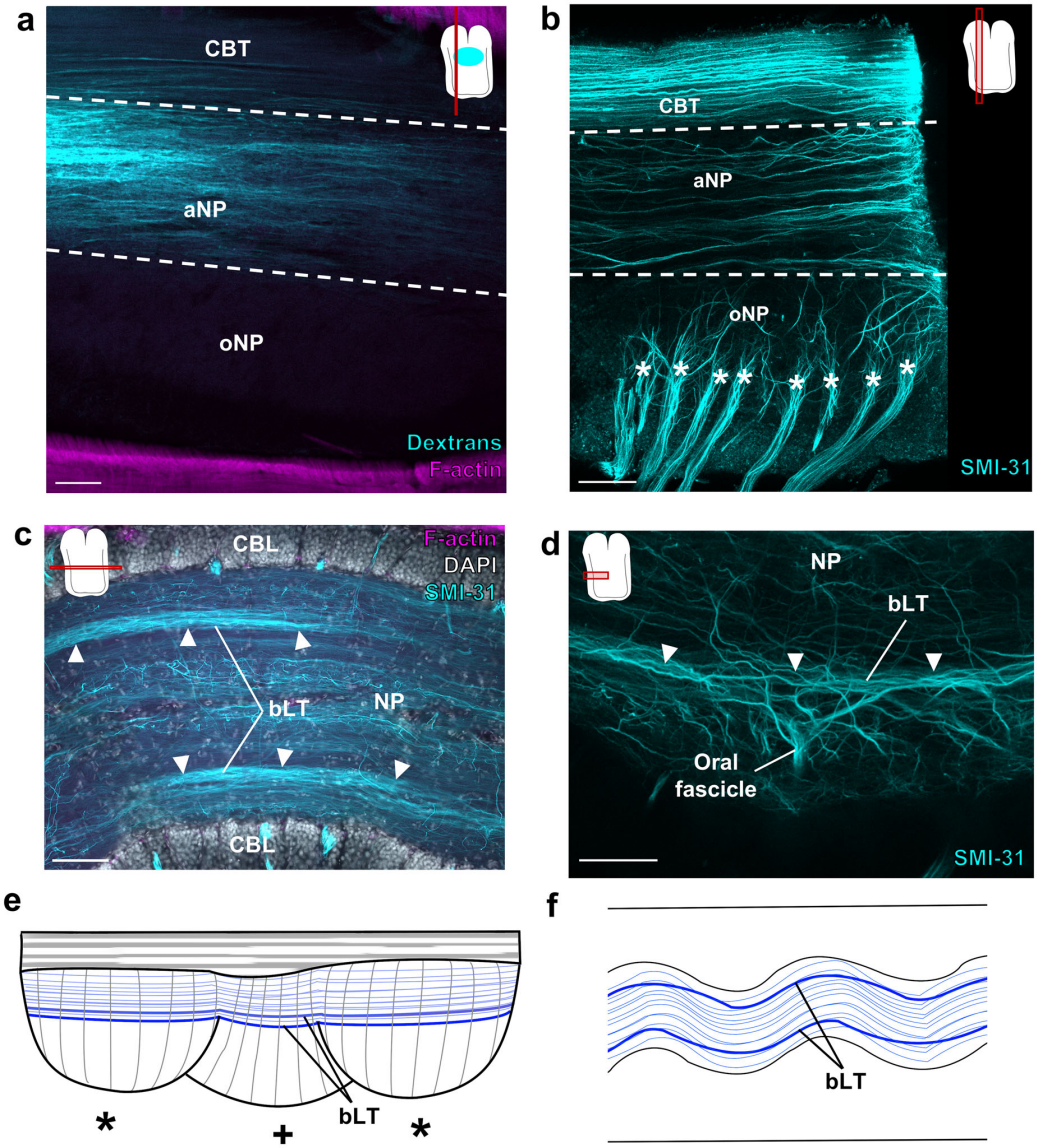
Longitudinal tracts in the aboral neuropil. (a, b) The aboral neuropil (NP) is dominated by longitudinal fibers. (a) Longitudinal section of an injection of dextran (cyan) into the aboral NP demonstrating longitudinal fibers. Key displays plane of section. Oval on key denotes injection site. (b) Maximum projection of a longitudinal wholemount with SMI‐31 immunostaining (cyan) marks the longitudinal fibers in the aboral NP. The oral fascicles (denoted by *) that become the oral roots targeting the sucker are also readily visible in this preparation. (c) Horizontal section at the transition from the oral NP to the aboral NP stained for SMI‐31 (cyan), F‐actin (magenta), and DAPI (gray). There are two prominent bilateral longitudinal tracts (bLT), marked by white arrowheads. (d) Maximum projection of a horizontal wholemount stained for SMI‐31 (cyan) confirms that the bLT corresponds to the most aboral branch point of the oral fascicles. (e–f) Summary diagrams showcasing the longitudinal tracts (blue) in the aboral NP in a longitudinal ANC cartoon (e) and a horizontal ANC cartoon (f). The bLT are denoted by thicker lines, and, in (e), are offset to demonstrate the tract on the opposite side. * in (e) denote ANC enlargements for suckers on the same side, + denote ANC enlargements for suckers on the opposite side. Scale bars: 100 µm. aNP, aboral neuropil; bLT, bilateral longitudinal tract; CBL, cell body layer; CBT, cerebrobrachial tract; NP, neuropil; oNP, oral neuropil.

We next interrogated the ANC NP for longitudinally running tracts with DiI, dextran labeling, and immunohistochemistry. The aboral, brachial NP is broadly dominated by longitudinal fibers (Figure 4a,b). These longitudinal fibers form a prominent bilateral pair of tracts (bLTs) situated at the transition between the oral sucker NP and the aboral brachial NP (Figure 4c; see also Sugarman et al. 2026). These tracts are positioned at the most aboral branch point of the oral fascicles, which feed to the oral roots innervating the sucker (Figure 4d; Olson, Schulz, et al. 2025). By their location at the interface between sucker and brachial territories of the ANC, these tracts could integrate motor control information coordinating the brachial musculature and sucker movements.

**FIGURE 5:**
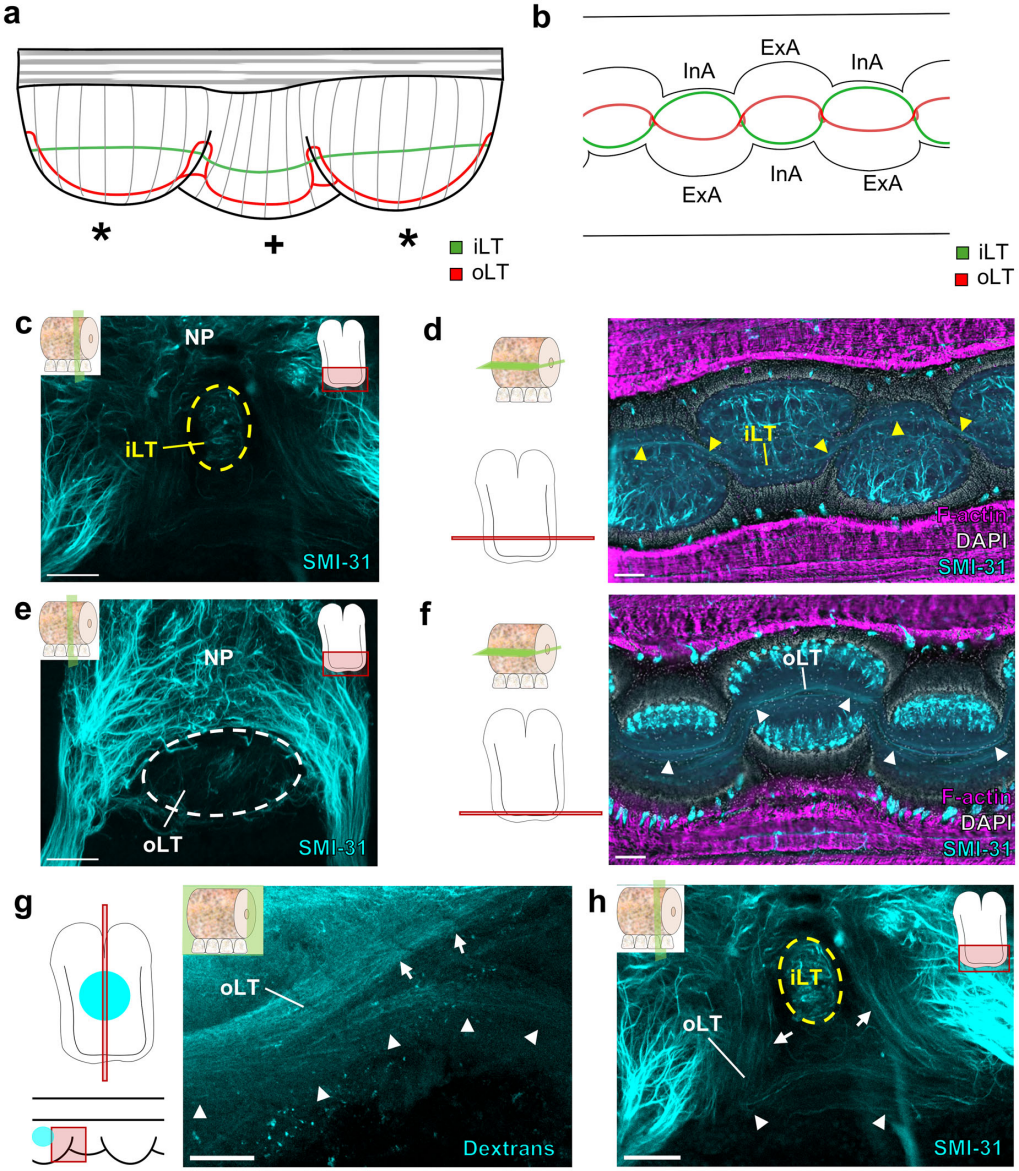
Longitudinal tracts in the oral neuropil. (a) Schematic of a longitudinal axial nerve cord (ANC) demonstrating the trajectories of the internal longitudinal tract (iLT, green) and the oral longitudinal tract (oLT, red) as the tracts travel from sucker to sucker. * denotes ANC enlargements for suckers on the same side. + denotes ANC enlargement for suckers on the opposite side. (b) Diagram of a horizontal section of the ANC demonstrating the paths of the oLT (red) and iLT (green) in the oral portion of the ANC. (c) Maximum projection of a transverse ANC wholemount stained for SMI‐31 (cyan). The iLT is outlined with a yellow dashed line as it crosses the midline. Key indicates transverse plane of section. Scale bar: 100 µm. (d) Horizontal section through the oral NP stained for SMI‐31 (cyan), F‐actin (magenta), and DAPI (gray). The iLT, marked by yellow arrowheads, follows the internal side of the ANC. (e) Maximum projection of a transverse ANC wholemount stained for SMI‐31 (cyan). The oLT is outlined with a white dashed line. Key indicates transverse plane of section. Scale bar: 100 µm. (f) Horizontal section through the oral NP stained for SMI‐31 (cyan), F‐actin (magenta), and DAPI (gray). The oLT, marked by white arrowheads, is positioned close to the midline, located oral to the iLT, and oscillates through ANC enlargements. Keys indicate horizontal plane of section. Scale bar: 150 µm. (g, h) The oLT bifurcates at the transition between sucker enlargements. White arrowheads mark the oral branch of the oLT, and white arrows mark the aboral branch of the oLT. See loop of the oLT in (a). (g) Injection of dextran labeling the oLT at the transition between sucker enlargements. Keys indicate plane of section and field of view. Ovals on keys indicate injection site. (h) Maximum projection of a transverse ANC wholemount stained for SMI‐31 (cyan) at the transition between sucker enlargements. The iLT is outlined with a yellow dashed line. The aboral oLT trunk loops over the iLT. The aboral and oral oLT branches connect to the oLT on the other side. Scale bars: 100 µm. ANC, axial nerve cord; ExA, external side of the axial nerve cord; InA, internal side of the axial nerve cord; iLT, internal longitudinal tract; NP, neuropil; oLT, oral longitudinal tract.

Within the oral sucker NP, we detected an internal longitudinal tract (iLT; Figure 5c,d) and an oral longitudinal tract (oLT; Figure 5e,f). The trajectories of the iLT and oLT are complex, and Figure 5a,b summarize our conclusions about their shifting relationships. In the horizontal plane, the iLT follows the internal side of the ANC, which corresponds to the internal side of the sucker (Olson, Schulz, et al. 2025; Olson, Moorjani, et al. 2025), switching from one edge to the other to maintain its relative positioning (Figure 5b,d). The oLT is located oral to the iLT and sits close to the center of the oral sucker NP (Figure 5a,e). As seen in horizontal sections, the oLT oscillates through the sucker enlargements (Figure 5b,f). As the ANC transitions to the next sucker enlargement, the oLT bifurcates into two trunks (Figure 5g,h). One of the oLT trunks ascends aborally to loop over the iLT, and the other continues orally (Figure 5g,h). Both branches connect directly to the oLT in the next sucker enlargement (Figure 5h). By projecting aborally, the oLT could receive input from other parts of the arm and mark the transition from one sucker to the next.

### 3.3 Comparative Analysis of Longitudinal Tracts

**FIGURE 6:**
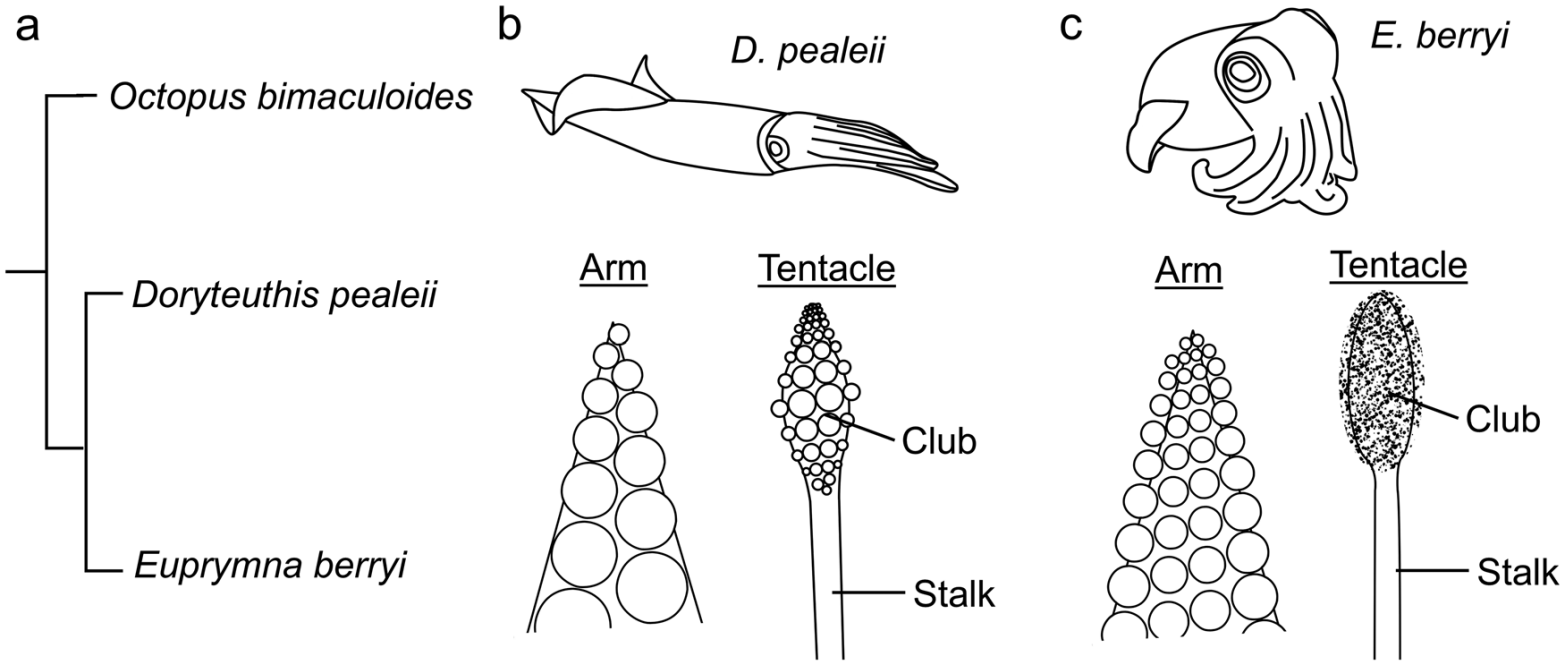
Phylogeny and gross morphology of arms and tentacles in *D. pealeii* and *E. berryi*. (a) Tree demonstrating the relationship between *Octopus bimaculoides* and two species of squid, *Doryteuthis pealeii* and *Euprymna berryi*. Octopodiforms and decapodiforms split ∼270 million years ago (mya); *D. pealeii, E. berryi* ∼100 mya. (b) Morphology of *D. pealeii* arm and tentacle. The arm is lined with two rows of suckers, the tentacle stalk is devoid of suckers, and the tentacle club is enriched in suckers similar to the suckers on the arm. (c) Morphology of *E. berryi* arm and tentacle. The arm is lined with 4 rows of suckers. The tentacle stalk is devoid of suckers. The tentacle club is lined with hundreds of small suckers.

We carried out a comparative analysis to assess the organization of ANC longitudinal tracts across coleoid cephalopods. We selected two species of squid for our analysis: The longfin inshore squid, *D. pealeii*, and the hummingbird bobtail squid, *E. berryi* (Figure 6). These squid species, which diverged from octopuses 270 million years ago (mya; Albertin et al. 2022), have eight arms and two tentacles. Like the arms of *O. bimaculoides*, these arms and tentacles are muscular hydrostats (Kier and Smith 1985; Kier 2016). In both squid species, the tentacles are utilized in a specialized prey capture behavior: The sucker‐poor tentacle stalks rapidly elongate to grab prey with the sucker‐rich tentacle clubs (Hanlon and Messenger 2018). Despite these similarities, *D. pealeii* and *E. berryi*, which diverged approximately 100 mya (Albertin et al. 2022), demonstrate key differences. *D. pealeii* are pelagic animals, found in the open ocean, whereas *E. berryi*, which are smaller animals, are benthic like *O. bimaculoides*. Additionally, the arms of *D. pealeii* have two rows of suckers (Figure 6b), whereas the arms of *E. berryi* are lined with four rows of suckers (Figure 6c). The tentacle clubs in *D. pealeii* are enriched with suckers similar in structure to the arm suckers, and, at the widest point, are arranged in rows of four suckers (Figure 6b). In contrast, the clubs of *E. berryi* are covered in hundreds of minute suckers with long, tube‐like stalks, which bear a resemblance to the tube feet of sea urchins (Figure 6c; see also Figure 10a; Florey and Cahill 1977).

**FIGURE 7:**
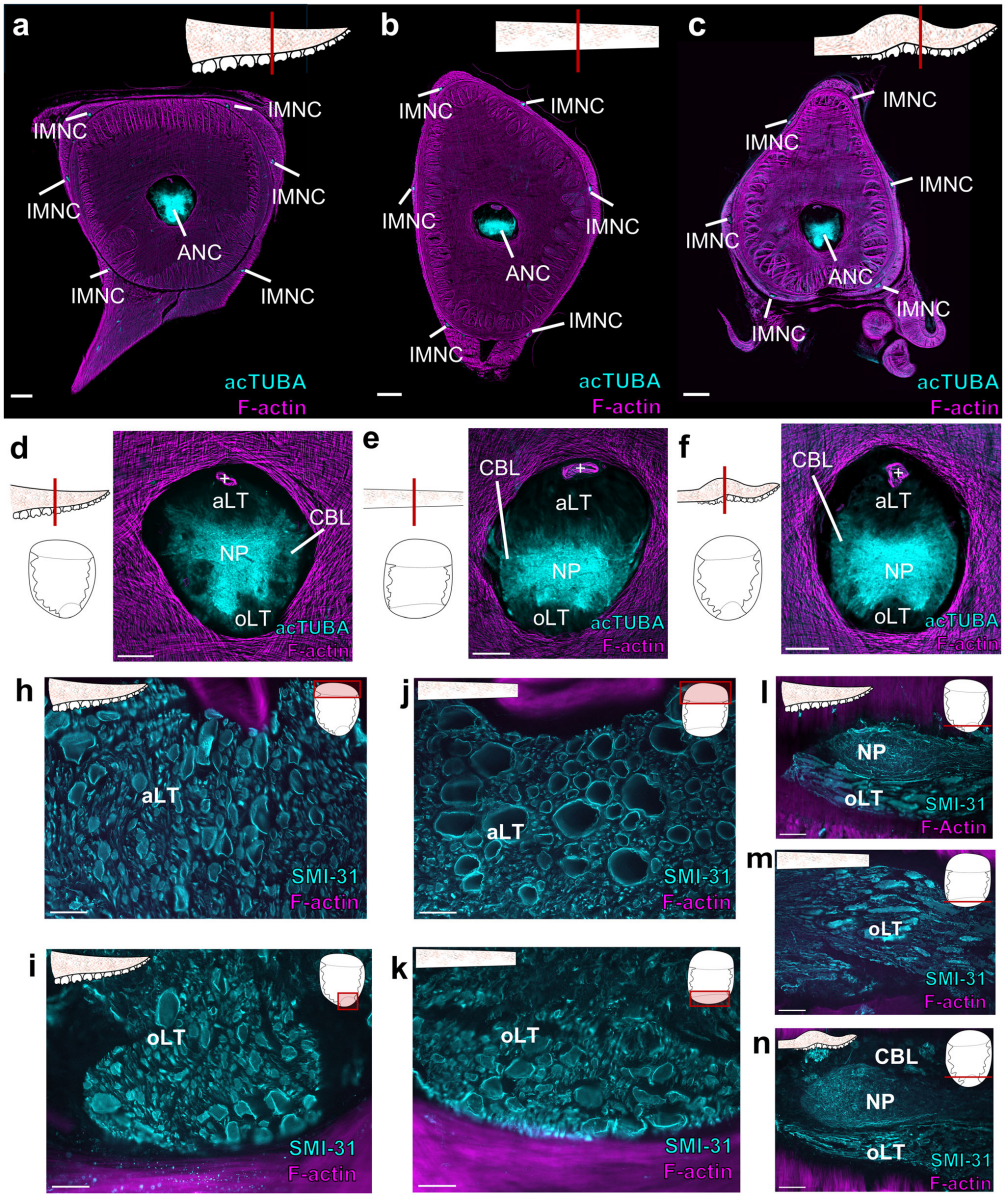
Organization of *D. Pealeii* arm and tentacle nervous system. (a–c) Transverse section of the *D. pealeii* arm (a), tentacle stalk (b) and tentacle club (c) stained for acTUBA (cyan) and F‐actin (magenta). The main components of the nervous system are highlighted. Scale bars: 500 µm. (d–f) Transverse section of the axial nerve cord in the *D. pealeii* arm (d), tentacle stalk (e) and tentacle club (f) stained for acTUBA (cyan) and F‐actin (magenta). Each ANC has an aboral longitudinal tract (aLT) and an oral longitudinal tract (oLT). + denotes brachial artery. Scale bars: 250 µm. (h–k) The aLT and oLT are composed of large fibers. (h, i) Transverse sections through the aLT (h) and oLT (i) in arm ANC stained for SMI‐31 (cyan) and F‐actin (magenta). (j, k) Transverse sections through the aLT (j) and oLT (k) in the tentacle stalk ANC stained for SMI‐31 (cyan) and F‐actin (magenta). Scale bars: 50 µm. (l–n) Horizontal sections through oLT in the arm (l), tentacle stalk (m) and tentacle club (n) stained for SMI‐31 (cyan) and F‐actin (magenta). In the arm (l) and tentacle club (n), where there are suckers, the oLT conspicuously weaves around neuropil (NP). Scale bars: 100 µm. acTUBA, acetylated α‐tubulin; aLT, aboral longitudinal tract; ANC, axial nerve cord; CBL, cell body layer; IMNC, intramuscular nerve cord; NP, neuropil; oLT, oral longitudinal tract.

The ANC of *D. pealeii*: Like *O. bimaculoides*, *D. pealeii* has an extensive nervous system embedded in the arms and tentacles. In addition to a prominent ANC, the arms and tentacles have six intramuscular nerve cords (IMNCs) distributed around the lateral edges (Figure 7a–c). The arms and tentacle clubs, which have suckers, also have sucker ganglia that, unlike *O. bimaculoides*, have a canonical ganglionic organization with cell bodies wrapping around NP (Richter et al. 2010; see Olson, Moorjani, et al. 2025). As noted in Olson, Schulz, et al. (2025), the ANCs in *D*. *pealeii* do not have a clear CBT equivalent to the octopus CBT, but do have major longitudinally running tracts distributed aborally and orally (Figure 7d–f; Guérin 1908). Fibers in the aboral longitudinal tract (aLT) and the oLT show a range of large diameters in transverse cross sections (Figure 7h–k; Guérin 1908). Those in the tentacle stalk aLT are the largest (Figure 7j). Both the aLT and the oLT are situated outside of the NP. In the arm and tentacle club, where there are suckers, the oLT oscillates around the NP (Figure 7l,n). In the tentacle stalk, the oLT runs straight (Figure 7m). We did not detect clear spatial subdivisions within the oLT or aLT as we did in the octopus CBT.

**FIGURE 8:**
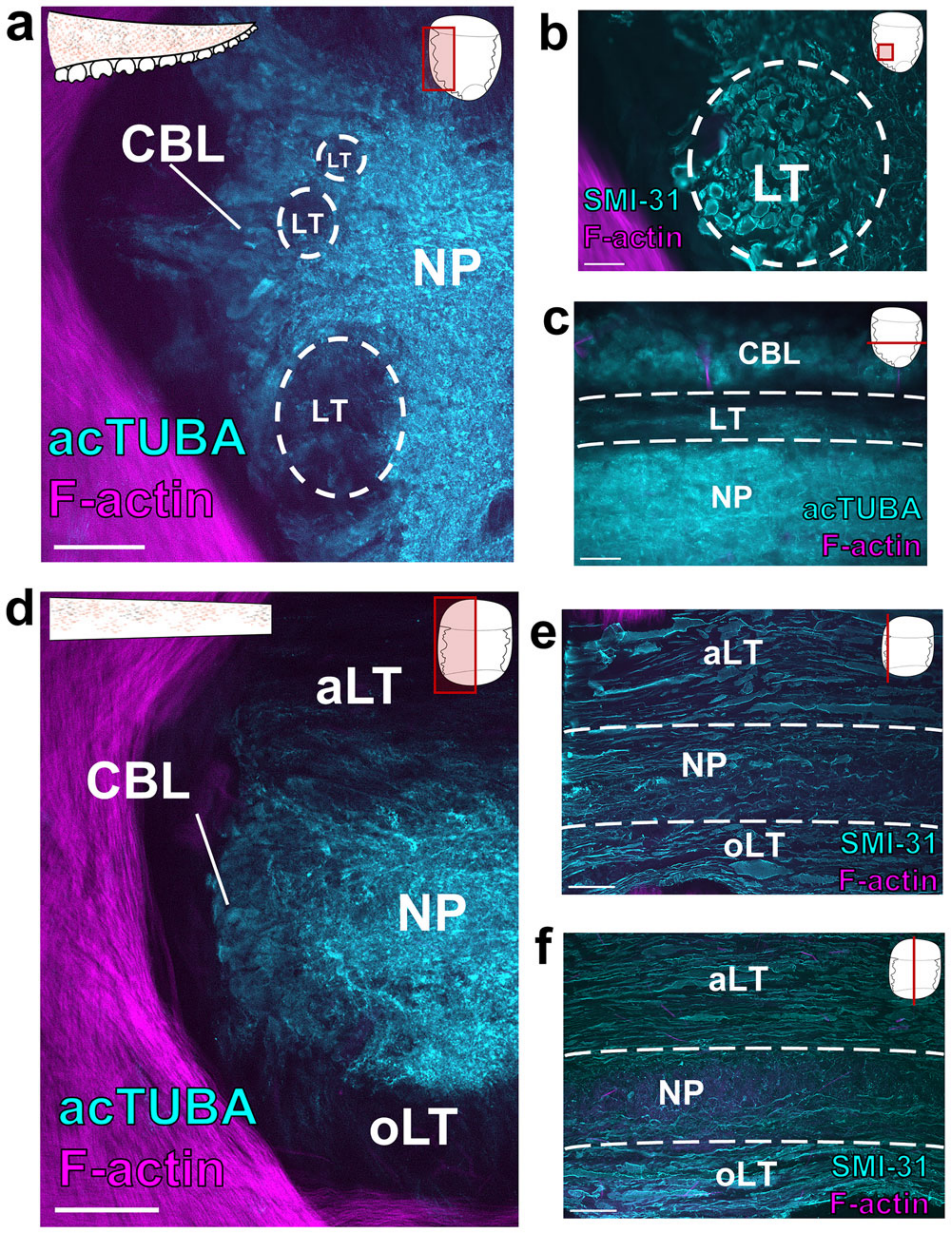
Longitudinal tracts in *D. pealeii* neuropil. (a) Transverse section through the axial nerve cord (ANC) in the squid arm stained for acetylated α‐tubulin (acTUBA, cyan) and F‐actin (magenta). Clear longitudinal tracts (LT), marked with white dashed line, are apparent by a decrease in acTUBA labeling. Inset depicts field of view. Scale bar: 100 µm. (b) Transverse section through the *D. pealeii* arm ANC with SMI‐31(cyan) immunostaining labels the fibers in the longitudinal tracts. Scale bar: 50 µm. (c) Horizontal section through the *D. pealeii* arm ANC stained for acTUBA. The longitudinal tracts are located at the border of the cell body layer (CBL) and neuropil (NP). Scale bar: 50 µm. (d) Transverse section through the ANC in the tentacle stalk stained for acetylated α‐tubulin (acTUBA, cyan) and F‐actin (magenta). There are no defined NP tracts. Scale bar: 100 µm. (e, f) Longitudinal sections through the tentacle stalk ANC stained for SMI‐31 (cyan) and F‐actin (magenta). Longitudinal fibers are abundant at the interface of the CBL and NP (e) but somewhat reduce in the center (f). Insets display plane of section. Scale bars: 100 µm. acTUBA, acetylated α‐tubulin; aLT, aboral longitudinal tract; ANC, axial nerve cord; CBL, cell body layer; LT, longitudinal tract; NP, neuropil; oLT, oral longitudinal tract.

We identified additional longitudinal fibers in the NP of the *D. pealeii* ANC (Figure 8). In the arm, these longitudinal fibers coalesce into distinct tracts (LT) situated at the interface of the CBL and NP (Figure 8a–c). The tentacle stalk ANC NP does not have distinct longitudinal NP tracts (Figure 8d). Instead, the entire border between the CBL and NP is lined with longitudinal fibers (Figure 8e,f). Having distinct bundles in *D. pealeii* arm but not tentacle could reflect differences in the sensorimotor demands for the suckers or the need to coordinate sucker and arm movements along a distance.

**FIGURE 9:**
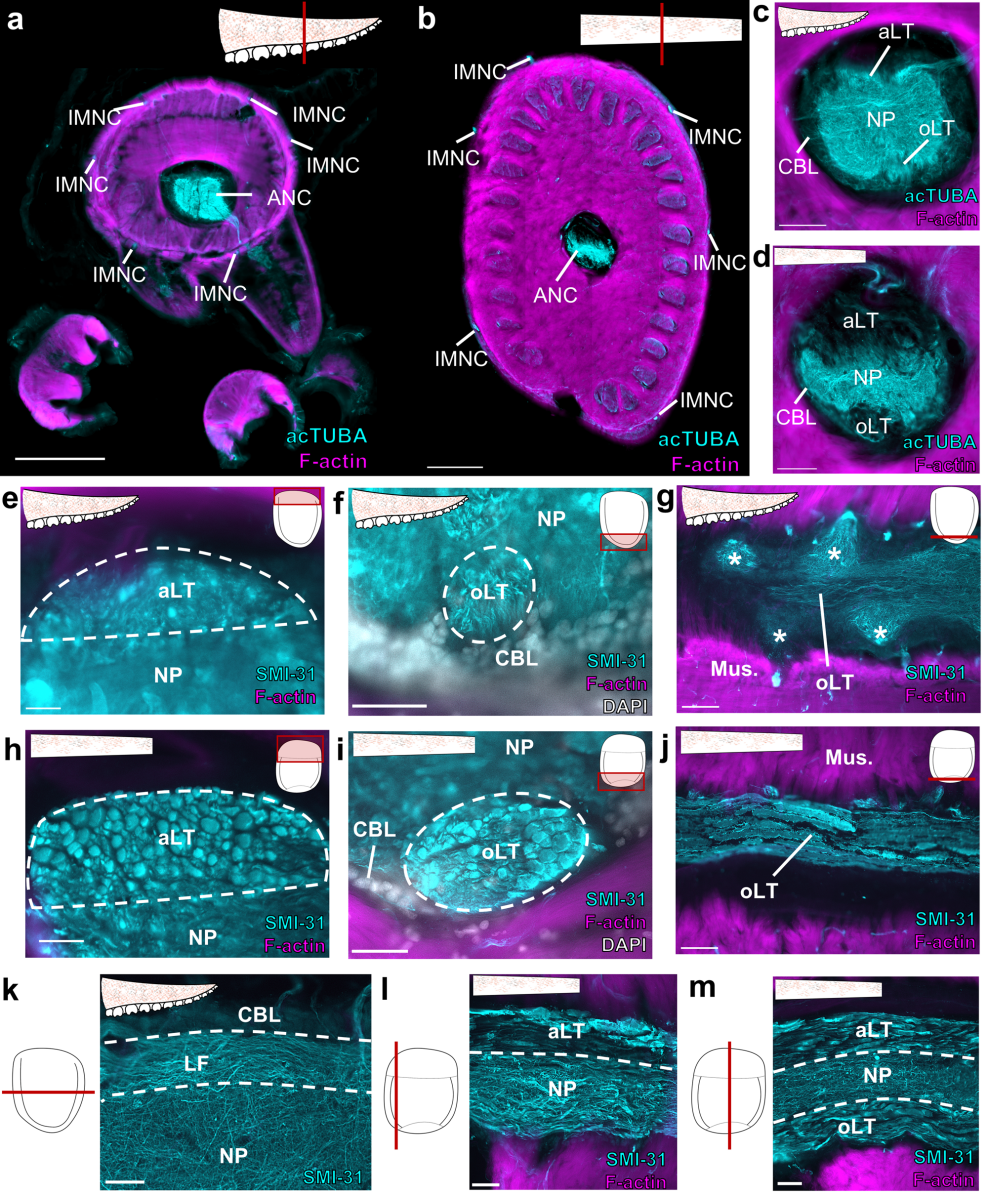
Organization of *E. berryi* arm and tentacle stalk nervous system. (a, b) Transverse section of the *E. berryi* arm (a) and tentacle stalk (b) stained for acTUBA (cyan) and F‐actin (magenta). The main components of the nervous system are highlighted. Scale bars: 500 µm. (c, d) Transverse section of the axial nerve cord (ANC) in the *E. berryi* arm (c) and tentacle stalk (d) stained for acTUBA (cyan) and F‐actin (magenta). Each ANC has an aboral longitudinal tract (aLT) and an oral longitudinal tract (oLT). Scale bars: 250 µm. (e, f) The aLT and oLT in the *E. berryi* arm are composed of small fibers. Transverse sections through the aLT (e) and oLT (f) in arm ANC stained for SMI‐31 (cyan), F‐actin (magenta), and DAPI (gray). Notably, the arm oLT is encompassed by the cell body layer of the ANC. Scale bars: 50 µm. (g) Horizontal section through the oLT in the arm ANC stained for SMI‐31 (cyan) and F‐actin (magenta). The oLT runs straight through sucker enlargements, denoted by *. Scale bar: 100 µm. (h, i) The aLT and oLT in the tentacle stalk are composed of large fibers. Transverse section through the aLT (h) and the oLT (i) in the tentacle stalk ANC immunostained for SMI‐31 (cyan), F‐actin (magenta), and DAPI (gray). The oLT in the tentacle stalk is not within the cell body layer. Scale bars: 50 µm. (j) Horizontal section through the oLT in the tentacle stalk ANC stained for SMI‐31 (cyan) and F‐actin (magenta). The oLT runs straight. Scale bar: 100 µm. (k–m) Longitudinal fibers in the NP of the arm and tentacle stalk ANC are abundant at the interface of the CBL and the NP. (k) Horizontal section through the arm ANC stained for SMI‐31 (cyan). The longitudinal fibers (LF) are located at the border of the cell body layer and neuropil. Key displays plane of section. Scale bar: 50 µm. (l, m) Longitudinal sections through the tentacle stalk ANC immunostained for SMI‐31 (cyan) and F‐actin (magenta). Longitudinal fibers are abundant at the interface of the CBL and NP (l) but reduce in the center (m). Key displays plane of section. Scale bars: 100 µm. acTUBA, acetylated α‐tubulin; aLT, aboral longitudinal tract; ANC, axial nerve cord; CBL, cell body layer; IMNC, intramuscular nerve cord; LF, longitudinal fibers; NP, neuropil; oLT, oral longitudinal tract.

**FIGURE 10:**
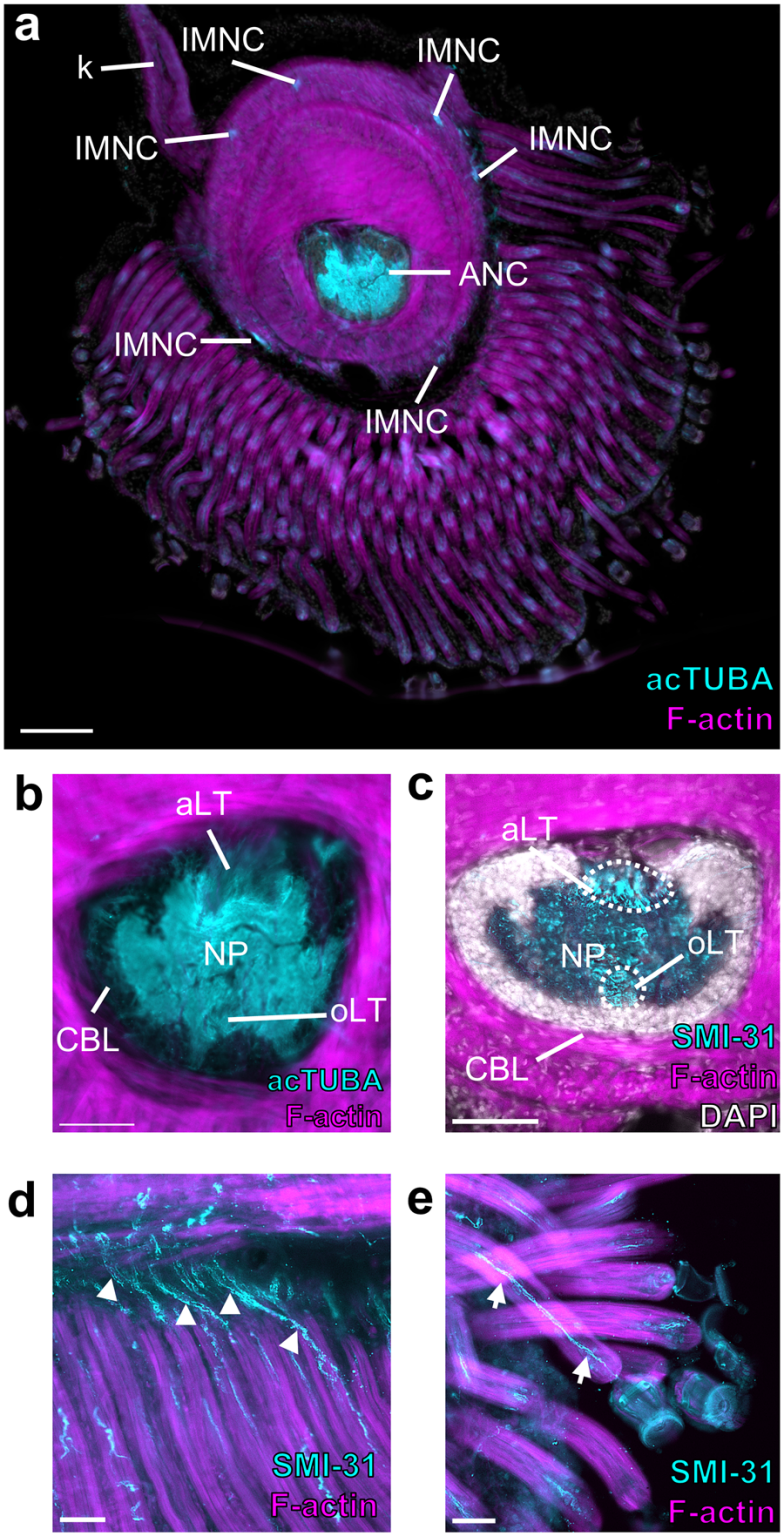
Organization of the *E. berryi* tentacle club nervous system. (a) Transverse section of the *E. berryi* tentacle club stained for acetylated α‐tubulin (acTUBA; cyan) and F‐actin (magenta). The main components of the nervous system are highlighted. The tentacle club is lined with minute suckers at the end of long tube‐like stalks. Scale bar: 500 µm. (b) Transverse section of the axial nerve cord (ANC) in the *E. berryi* tentacle club stained for acTUBA (cyan) and F‐actin (magenta). The neuropil (NP) is expanded and the aboral longitudinal tract (aLT) appears compressed. (c) Transverse section of the ANC stained for SMI‐31 (cyan), F‐actin (magenta), and DAPI (gray). The aLT and the oral longitudinal tract (oLT) are composed of small fibers, and the oLT is within the NP. Scale bars: 250 µm. (d, e) The ANC issues nerves that travel down the long tube‐like sucker stalks to target the minute suckers. (d) Longitudinal section through the tentacle club stained for SMI‐31 (cyan) and F‐actin (magenta). White arrowheads point to ANC nerves as they begin to travel along the sucker stalks. (e) Longitudinal section through the tentacle club stained for SMI‐31 (cyan) and F‐actin (magenta). White arrows indicate an ANC nerve traveling along the sucker stalk to target the sucker. Scale bars: 25 µm. acTUBA, acetylated α‐tubulin; aLT, aboral longitudinal tract; ANC, axial nerve cord; CBL, cell body layer; IMNC, intramuscular nerve cord; k, swimming keel; NP, neuropil; oLT, oral longitudinal tract.

The ANC of *E. berryi*: In the arms and tentacles of *E. berryi*, there is a prominent ANC mediating sensorimotor control (Figure 9a–d, Figure 10a,b). Even though the arm is lined with four rows of suckers, the ANC innervates each sucker separately, and each sucker has a dedicated sucker ganglion (Figure 9a). *E. berryi* arms and tentacle stalks also exhibit six IMNCs (Figure 9a,b; Figure 10a).

Within the arm of *E. berryi*, the ANC has a discernable aLT (Figure 9c,e) and oLT (Figure 9c,f). The aLT is situated outside of the NP (Figure 9e), whereas the oLT is within the NP (Figure 9f). Both tracts are composed of small fibers. The oLT lies close to the midline and runs straight while enlargements for the suckers oscillate around it (Figure 9g). Beyond the oLT, we failed to identify additional defined NP tracts. However, we found that longitudinal fibers line the interface of the CBL and the NP (Figure 9k). With an extra‐NP aboral tract and an intra‐NP oral tract, the arrangement of the longitudinal tracts in the *E. berryi* arm ANC is reminiscent of those in the ANC of *O. bimaculoides*.

The tentacle stalk ANC of *E. berryi* likewise contains an aLT and oLT (Figure 9d,h,i). Both tracts are composed of large fibers and are situated outside of the NP (Figure 9h,i). This arrangement is similar to the distribution of longitudinal tracts in the tentacle stalk ANC in *D. pealeii*. As in the *D. pealeii* tentacle stalk ANC, the oLT runs straight (Figure 9j). We examined the NP of the ANC in the tentacle stalk of *E. berryi* for additional longitudinal tracts. While we failed to uncover any distinct longitudinal tracts, the border of the CBL and NP contains many longitudinal fibers (Figure 9l,m).

Despite its unique morphology, the tentacle club in the *E. berryi* maintains major features of the cephalopod appendage nervous system: A prominent ANC with IMNCs distributed laterally (Figure 10a). Compared with tentacle stalk, we found that the club ANC exhibits an expanded NP (Figure 10b). The aLT appears compressed, and the oLT is greatly diminished (Figure 10b,c). In major contrast with that of the tentacle stalk, the oLT is within the NP and is populated with smaller fibers (Figure 10c). This composition of the oLT is similar to that of the *E. berryi* arm. This suggests that, within *E. berryi*, whether the ANC innervates suckers may dictate the positioning of the oLT. Indeed, the ANC in the tentacle club issues nerves that target the hundreds of minute suckers lining the club (Figure 10d,e). The unique sensory and motor demands of this array of fine suckers warrant further study.

## 4 Discussion

Many of the complex behaviors shown by the octopus require the coordination of suckers and arm musculature along the length of the arm (Kier and Smith 1985; Gutfreund et al. 1998; Sumbre et al. 2006; Kier 2016; Hanlon and Messenger 2018; Kennedy et al. 2020; Bidel et al. 2022). Accordingly, the ANC in *O*. *bimaculoides* hosts many longitudinal fiber tracts that are strong candidates to support these behaviors. Some of these behaviors involve coordination of suckers along the proximal‐distal axis, such as passing food along the arm according to its valence (Altman 1971). The oLT and the α tract in the CBT may serve as a substrate for sensorimotor communication underlying these behaviors. Sucker movements often recruit the movement of the rest of the arm, whether it is to extend or rotate the arm to grasp an object or to use the arm to push an object away (Sumbre et al. 2006; Kennedy et al. 2020). The bilateral tracts situated in the brachial NP, just aboral to the sucker NP, may serve as pathways for coordinating these behaviors. It is undoubtedly the case that the different arms, together with their suckers, can be coordinated in behaviors such as walking on the ocean floor or rotating a ball (Bennice et al. 2025; see supplemental video 1 in Olson, Schulz, et al. 2025). At least some of this is likely mediated by ANC‐to‐ANC connections traveling across arms by way of the interbrachial commissure (Graziadei 1971; Young 1971; Chang and Hale 2023). Classic studies suggest that the interbrachial commissure is formed in part by longitudinal fibers from the NP of the ANC (Graziadei 1971). Further studies are required to determine whether these longitudinal fibers arise from the NP tracts that we define here and how the longitudinal tracts in the NP interconnect with the interbrachial commisure and the brain (see Sugarman et al. 2026).

We confirmed the existence of the longitudinal tracts in the octopus arm nervous system with four different techniques— two tracing methods and two antibodies. Our results demonstrate that two features drive the organization of the longitudinal tracts: Whether they are intra‐ NP tracts, such as the iLT, or extra‐ NP tracts, like the CBT, and whether they pertain to the sucker or to the brachial musculature. Within the CBT, the α tract appears to be strongly coupled with the sucker, whereas the γ and δ tracts have connections particularly to the brachial territory of the ANC. The four defined intra‐NP tracts (oLT, iLT, bLTs) are either situated within the sucker territory of the ANC or demonstrate clear links to the oral roots that innervate the sucker. Although the brachial territory of the ANC contains longitudinal fibers, it lacks defined tracts clearly dedicated to the brachial musculature. Given its proximity to CBT, the defined tracts within the CBT, such as the γ and δ tracts, could be used as intermediate relays for the brachial musculature. Nerves arising from the brachial territory of the ANC also connect to the skin covering the arm (Olson, Schulz, et al. 2025). Prior research suggests that efferent fibers from the chromatophore lobes in the brain directly connect to the arm chromatophores (Young 1971). These fibers most certainly travel through the CBT before descending into the brachial NP to join the nerves innervating the skin. Additional studies of how the subtracts of the CBT interconnect with the brain would reveal the pathways used by chromatophores’ efferent fibers.

In the vertebrate nervous system, spinal cord tracts are divided into sensory tracts and motor tracts. Previous work in the octopus arm nervous system demonstrates that broad segregation of sensory and motor neuron cell bodies is not a principle feature of the ANC CBL and suggests that ANC nerves are mixed, carrying both sensory and motor information (Rossi and Graziadei 1958; Rowell 1966; Gutfreund et al. 2006; Olson, Schulz, et al. 2025; Olson, Moorjani, et al. 2025). The longitudinal tracts within the ANC could be subdivided into separate bundles for sensory and motor information, providing a method for unmixing sensory and motor signals. Functional studies are needed to assess this possibility. Physiological studies have shown that mechanosensory signals, at the least, travel both proximally and distally along the ANC (Gutfreund et al. 2006; Chang and Hale 2023). This aligns with our tract‐tracing results, which demonstrate proximal and distal connections to the longitudinal tracts.

The comparisons to squid demonstrate that despite differences in appendage morphology, environmental context and behavioral needs, there are shared features in ANC longitudinal tract structure. Across the cephalopod species studied, all ANCs contain an aLT (CBT in *O. bimaculoides*) located outside of the NP, and an oLT. With its two trunks and readily observable subdivisions, the CBT in *O. bimaculoides* demonstrates a more complex organization when compared with the aLT in squid, which could underlie an increase in behavioral complexity and sensory function (Albertin et al. 2022; Kang et al. 2023). The oLT in the *D. pealeii* arm is composed of large fibers and is situated outside of the NP, whereas the oLT in the *O. bimaculoides* and *E. berryi* arms is solidly within NP. This displacement of the oLT in *D. pealeii* suggests a bias towards longer distance connections. Whether the oLT or the aLT in squid correspond to the CBT by interconnecting with the brain needs experimental testing with tract‐tracing methods.

The organization of the ANC within the tentacle stalk appears remarkably conserved between *D. pealeii* and *E. berryi*. In each, the oLT and aLT are extra‐NP tracts, composed of fibers with large diameters. Additional longitudinal fibers dominate the interface between the CBL and NP. This similarity in neural structure is underscored by similarities of the behavioral output in tentacle stalks (Kier and Smith 1985; Kier 2016). The tentacle stalks elongate rapidly to grab prey, reaching their target in 15–35 ms in loliginid squid (Kier 1985). With the speed of this motor act, the main requirement of the tentacle stalk ANC could simply be to propagate a signal from the brain along the length of the stalk and to relay sensory feedback from the clubs to the brain. In the absence of myelin, large nerve fibers in the aLT and oLT within the stalk ANC seem well suited for fast transmission of a neural signal.

The longitudinal tracts in the ANCs of octopus and squid arms exist in the context of a clear segmental organization of the ANC CBL (Olson, Schulz, et al. 2025). Neither the intra‐NP nor extra‐NP tracts themselves are segmented, but, by their positioning, the intra‐NP tracts would likely receive modulation from the neurons situated in the segments. The CBT, as an extra‐NP tract, may serve as an unmodulated relay of sensorimotor commands. Further descriptions of the anatomical and functional inputs to the intra‐NP and extra‐NP tracts would clarify how neural signals are transformed along the tracts.

## 5 Conclusion

Our findings point to a hierarchy within the arm nervous system for sensorimotor control of the suckers and the brachial musculature. First, each sucker houses a sucker ganglion, a neural center well‐ situated for local sensorimotor integration for the sucker, with relays to the oral ANC (Olson, Moorjani, et al. 2025). The ANC contains enlargements for each sucker, which are subdivided into segments to create a topographic map, or “suckerotopy”, for each sucker (Olson, Schulz, et al. 2025). This establishes a further level of sensorimotor control. Next, as described in this study, multiple complex longitudinal circuits within the NP interconnect each ANC enlargement to the next, allowing for coordinated movements across the suckers and brachial musculature. Finally, there is the CBT, the massive octopus aboral fiber tract, which provides for longer distance intra‐arm connections for both the sucker and the brachial NP. This hierarchy may be key for understanding how the interbrachial connectives and intermediate motor areas within the brain mediate sensorimotor control for octopus arm movements, from simple deflections of individual suckers to complex, coordinated whole body actions.

## Conflicts of Interest

The authors declare no conflicts of interest.

## Data Availability

Data available upon request.
